# Medial sural artery perforator free flap for small- to medium-sized defects in head and neck reconstruction: a suitable replacement for radial forearm free flap

**DOI:** 10.1186/s40902-024-00455-4

**Published:** 2025-02-08

**Authors:** Yulian Zhang, Keran Pan, Jian Wu, Xi Tang

**Affiliations:** 1 Department of Head and Neck Oncology, Chongqing University Cancer Hospital, Chongqing, China; 2 Chongqing Key Laboratory for Intelligent Oncology in Breast Cancer (iCQBC), Chongqing University Cancer Hospital, Chongqing, China; 3 Cancer Center, Faculty of Health Sciences, University of Macau, Macau SAR, China

**Keywords:** Medial sural artery perforator flap, Head and neck, Reconstruction, Donor-site morbidity

## Abstract

**Background and objectives:**

To compare clinical outcomes and donor site morbidity between medial sural artery perforator (MSAP) flap and radial forearm free (RFF) flap for soft tissue reconstruction of head and neck.

**Methods:**

Forty-six patients who underwent free flap reconstruction at the head and neck cancer center from February 2019 to March 2021 were included, of which 25 RFF flaps and 21 MSAP flaps. The patient and flap characteristics (age, sex, flap size, harvest time, etc.) and outcomes (success rate, donor site complications including infection, hematoma, and fistula, donor site morbidity including abnormal sensation, weakness, range of motion, postoperative oral function) were recorded and compared. Patients were followed up for at least 12 months after surgery. The patients were assessed subjective donor-site morbidity and satisfaction with overall functional results using a self-reported questionnaire.

**Results:**

The success rates of RFF flaps and MSAP flaps were 96% and 95.2%. There were no significant differences in age, sex, flap size, pedicle length, postoperative treatment, and postoperative oral function. MSAP flap showed less donor site morbidity and better subjective satisfaction at the donor site than RFF flap did after a 12-month follow-up. A dominant perforator of the medial sural artery emerges constantly near the point which is approximately 15 cm from the popliteal fossa center vertically, and 3 cm from the postor midline of the leg horizontally.

**Conclusion:**

Due to less donor site morbidity and higher patient satisfaction, MSAP flap can be used as a replacement for RFF flap for small to medium-sized defects in head and neck reconstruction.

**Supplementary Information:**

The online version contains supplementary material available at 10.1186/s40902-024-00455-4.

## Introduction

Significant tissue defects are often seen after radical resection of head and neck cancer, especially if the cancer occurs in the oral cavity. In such cases, reconstruction surgery always requires flap repair to enable recovery of oral function. In the past three decades, radial forearm free (RFF) flap and anterolateral thigh (ALT) flap have been the best choices [1–3]. To date, the RFF flap was the most common flap for repairing post-oncologic defects. However, due to significant cosmetic and functional donor-site morbidity, and sacrifice of one major artery (radial artery) [4, 5], skillful microsurgeons tend to use perforator flaps. MSAP flap was first described about two decades ago and showed good results and minimal donor site morbidity. But its usage in the head and neck is rare, especially for the study comparing with RFF flap in head and neck reconstruction. We therefore conducted a study for 46 cases treated with MSAP flap and RFF flap to compare clinical outcomes and donor site morbidity of these two methods in head and neck reconstruction.

## Materials and methods

Forty-six cases of flap surgery were conducted for reconstructing small- to medium-sized defects in our department between February 2019 and March 2021. Patients were free to choose between being reconstructed with RFF flap or MSAP flap. There were 25 RFF flaps and 21 MSAP flaps. Patient’s age, sex, primary site, tumor stage, flap thickness, flap size, pedicle length, harvesting time of flap, outcomes, and donor site morbidity were reviewed. The subjective functional and cosmetic assessments of all the patients’ donor sites were recorded. To assess subjective function and cosmetic results, all patients were asked to rate postoperative results of donor-site from 1 to 100 on appearance, sensory disturbance, swelling, range of motion, weakness, and cold intolerance. A scale of 1–100 scores to evaluate the postoperative scores, where a preoperative score of donor sites is 100. We evaluate postoperative oral function by swallowing and speech, which are most related to flap types. The scale standards are cited from the University of Washington Quality of Life Questionnaire (UW-QOL version. 4) [6] as below (Table 1).

**Table 1:** University of Washington Quality of Life Questionnaire (UW-QOL version. 4)

Swallowing
I can swallow as well as ever. (100)
I cannot swallow certain solid foods. (70)
I can only swallow liquid food. (30)
I cannot swallow because it “goes down the wrong way” and chokes me. (0)
Speech
My speech is the same as always. (100)
I have difficulty saying some words but I can be understood over the phone. (70)
Only my family and friends can understand me. (30)
I cannot be understood. (0)

### Surgical techniques for RFF flap

An Allen test was performed, and main vessels were marked using Duplex-Doppler ultrasound before the operation. The radial forearm flap centered on the cephalic vein and radial artery was designed according to the defect size. The flap was cut along the outer edge of the flap to the superficial surface of the deep fascia and was lifted from distally to proximally on the surface of the myomembrane, and the cephalic vein was identified first. Careful ligation of the cephalic vein and suture it to the superficial tissue of the flap to avoid detaching of cephalic vein and flap. Then the radial vessels were ligated and dissected retrogradely; meanwhile, the aponeurosis with a microvascular network on the surface of the flexor carpal flexor and the radial flexor were preserved completely in order to keep the blood supply for skin graft. Elevate the flap retrogradely, with the radial vessels and cephalic vein included, and ligate the microvascular branches of radial vessels to muscles. Skeletonize the radial vessels and cephalic vein and obtain a long enough pedicle.

In this procedure, the median nerve and superficial branches of the radial nerve should not be damaged; the microvascular network on the surface of the flexor carpal flexor and the radial flexor were preserved. An abdominal full-thickness skin graft was performed for a secondary defect of the forearm after wound washing with static pressure dressings. The static pressure dressings will be released 7–10 days after surgery to check out the survival of the skin graft.

### Surgical techniques for MSAP flap

**Fig. 1:**
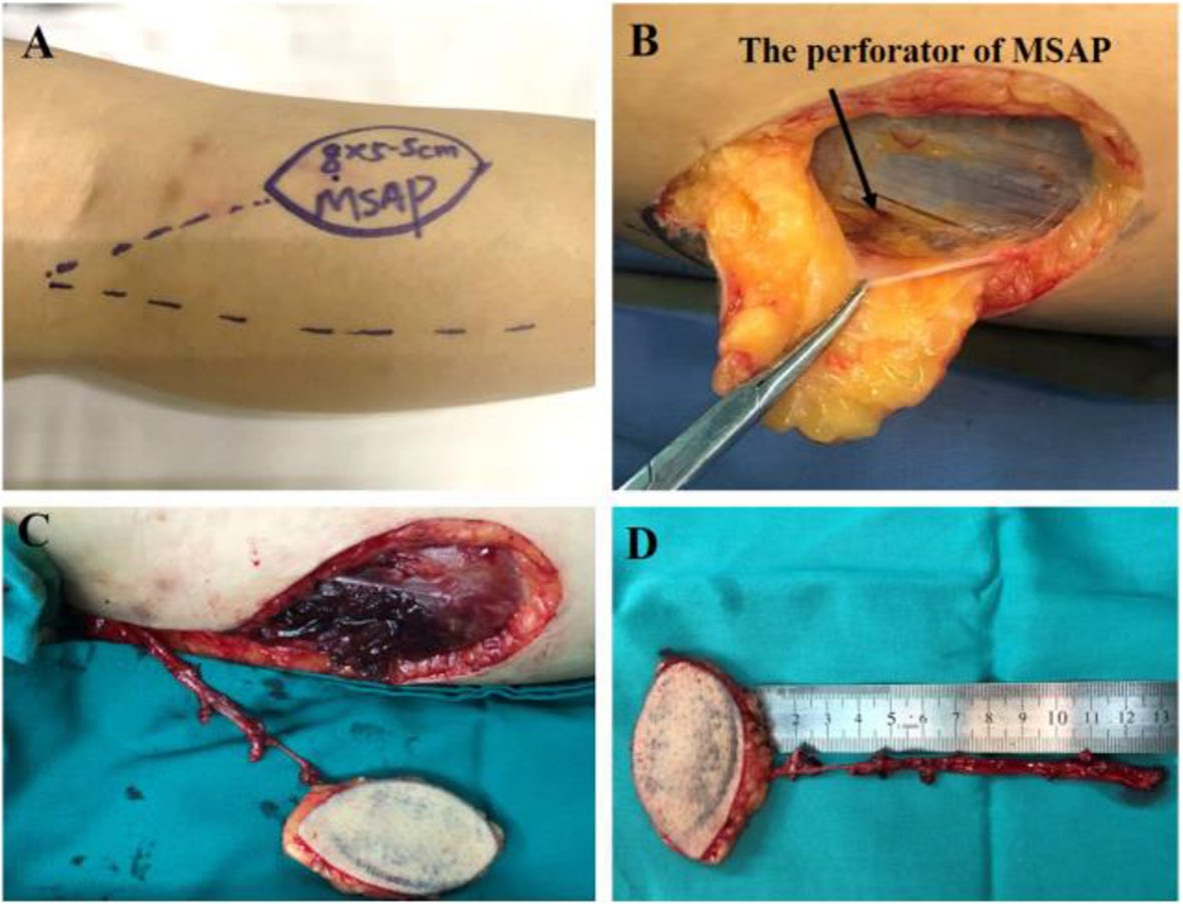
Dissection of MSAP flap. **A** Flap is outlined over the substance of medial gastrocnemius muscle, and the audible perforators are marked using Duplex-Doppler ultrasound. **B** Medial incision is carried out and the perforators are identified. **C** All the branches are clipped or cauterized. **D** The tourniquet is released to confirm circulation, then the flap is islanded

All dominant perforators were marked using Duplex-Doppler ultrasound, and the flap thickness was measured where the perforators emerged. Horizontal distances from the midline to the perforator and vertical distances from the popliteal fossa to the perforator were measured (Fig. 1A). We designed the flap according to the perforator location; the axis of the flap is the line between the popliteal crease to the perforator. We selected the non-dominant leg for the donor side, which was mostly the left leg. The patient was placed in the supine position with the hip abducted and externally rotated and the knee flexed. The operation bed was rotated a little to the donor site side. A tourniquet was used with the leg elevated to prevent exsanguination. This allowed the filling of the vessels, which helped in microvascular dissection.

The medial curve incision was made first. The flap was elevated above or under the deep fascia plane to identify the musculocutaneous perforators. Then, intramuscular dissection was performed to ensure that the perforators were available (Fig. 1B). A lateral incision was performed to elevate the flap, after which intramuscular dissection of the perforator was continued following the trunk of the medial sural artery and the side branches were ligated to the muscles (Fig. 1C). Dissection was continued until a sufficient length of the pedicle was available and the tourniquet was released to check blood supply of the flap. Finally, the pedicle was cut off and adapted to the recipient site. Close the donor site primarily with or without a drainage (Video. 1 Supplementary).

After the surgery was completed, patients were prescribed complete bed rest for 3–5 days. The blood supply of the flap was initially monitored once per hour.

### Statistical analysis

An independent *t*-test was used to test the null hypothesis, which stated that the means of the two groups were equal. Fisher’s exact test was used to analyze the differences in the group complication rates and surgical outcomes. All statistical tests were two sided, and a value of *p* < 0.05 was considered statistically significant. All statistical analyses were conducted using SPSS version 19.0.

## Results

There were 13 male and 8 female patients, and mean age is 57.1 (range, 39–79) years in the MSAP flap group, while 18 male and 7 female patients, mean age is 56.2 (range, 36–78) years in the RFF flap group. All the donor sites were on the left side, except in two cases in the MSAP flap, and all the donor sites were on the left side in the RFF group. All patients had oral squamous cell carcinoma. The most common site of tumor in the MSAP flap group was the tongue (12 cases); other sites were the floor of the mouth, buccal mucosa, and gingiva. In contrast, there were 15 cases of the tongue, 7 cases of buccal, and other primary sites. Most of the cases in the two groups were T1-2 stage according to the American Joint Committee on Cancer (AJCC) Tumor classification (Table 2).

**Table 2:** Demographics, tumor stage, primary site, and postoperative radiotherapy undergoing flaps for head and neck reconstruction

	Flap type		*P* value
	RFF flap	MSAP flap	
**Number**	25	21	
**Age (years)**	56.2 (36–78)	57.1 (39–79)	0.783
**Gender**			
Male	16	13	
Female	9	8	
**Tumor stage**			
T1	5	6	
T2	18	14	
T3	1	0	
T4	1	1	
**Primary site**			
Tongue	15	12	
Buccal	7	3	
Floor of mouth	2	3	
Base of tongue	0	1	
Gingiva	1	2	
**Postoperative radiotherapy received**	17	14	

### The MSAP flap to RFF flap have similar characteristics for reconstructing small- to medium-sized defects in the head and neck, but more time is needed for MSAP flap harvesting than RFF flap harvesting

Flap dimensions varied with a mean length of 7.33 (range 6–11) cm, width of 4.95 (range 4–7) cm, and thickness of 6.05 (range 4–10) mm. The mean pedicle length was 11.57 (range 9–16) cm in the MSAP flap group. Meanwhile, the mean length is 7.44 (range 5–10) cm, mean width is 5.12 (range 4–6) cm, and mean thickness is 5.28 (range 4–8) mm; the mean pedicle length was 10.64 (range 8–13) cm in the RFF flap group. Each of the MSAP flap carried only one perforator. Both groups suffered one flap total flap necrosis respectively. The one in the MSAP flap group is related to venous congestion on day 6th after surgery due to improper neck rotation. The one in the RFF flap group was also caused by venous congestion on day 2 after surgery, despite we had a second exploration surgery and reanastomosed the congested vein. All the donor sites primarily healed except 3 cases of delayed healing and without long-term complications in the MSAP flap group. All the donor sites had skin grafted in the RFF flap group. Four cases of skin graft suffered partial or total necrosis, and three cases suffered infection; seven cases received delayed healing in more than 3 weeks (Table 3). The average harvesting time of flaps is 46.05 min versus 30.92 min (MSAP vs. RFF) (*p* < 0.001). All the cases in the RFF flap group required skin graft in the donor site, while all the cases in the MSAP flap group were closed primarily (Table 4).

**Table 3:** Postoperative donor site complications and morbidity (12-month follow-up)

	MSAP flap	RFF flap
**Complications**		
Wound infection	0	3 (12%)
Hematoma	0	0
Fistula	0	0
Delayed heal	3	7 (28%)
Partial or total skin graft loss	0	4 (16)
**Morbidity**		
Limited ROM	0	7 (28%)
Weakness	0	3 (12%)
Abnormal sensation	0 (0/21)	100% (25/25)

**Table 4:** Data about flap characteristics

	Flap type		*P* value
	RFF flap	MSAP flap	
Flap length, cm			
Mean	7.44	7.33	0.787
Range	5 ~ 10	6 ~ 11	
Flap width, cm			
Mean	5.12	4.95	0.377
Range	4 ~ 6	4 ~ 7	
Flap thickness, mm			
Mean	5.28	6.05	0.052
Range	4 ~ 8	4 ~ 10	
Pedicle length, cm			
Mean	10.64	11.57	0.068
Range	8 ~ 13	9 ~ 16	
Flap harvest time, minutes			
Mean	30.92	46.05	< 0.001
Range	20 ~ 47	40 ~ 63	
Flap necrosis	1/25	1/21	

#### The location of dominant perforator of MSA is constant

All the dominant perforators were marked accurately. The dominant perforator is located 14.81 cm ± 1.23 from the popliteal fossa (PF) vertically and 3.24 cm ± 0.41 from the postor midline (PML)horizontally in the left leg, The dominant perforator is located 15.17 cm ± 1.03 from PF vertically and 3.07 cm ± 0.66 from PML horizontally in the right leg, The dominant perforator is generally located 14.99 cm ± 1.13 from PF vertically and 3.15 cm ± 0.55 from PML horizontally (Table 5).

**Table 5:** Data about the location of MSAP

	In left leg (cm)	In right leg (cm)	Both left and right legs (cm)
Vertical distance from perforator to PF	14.81 ± 1.23	15.17 ± 1.03	14.99 ± 1.13
Horizontal distance from perforator to PML of leg	3.24 ± 0.41	3.07 ± 0.66	3.15 ± 0.55

#### Both flaps have good postoperative oral functions, but the MSAP flap group has less donor-site morbidity

There were 17 cases in the RFF flap group and 14 cases in the MSAP flap group who received postoperative radiotherapy with a dosage of 50–56 Gy. The score of postoperative swallowing function in the two groups is 87.1 versus 84.4 ( MSAP vs. RFF, *p* = 00.547). The score of postoperative speech function in the two groups is 90 versus 86.8 ( MSAP vs. RFF,* p* = 0.471).

All the cases in the RFF flap group revealed an abnormal sensation associated with superficial branch of the radial nerve in the donor site. Three cases showed weakness in the donor site wrist. Seven cases complained of adhesion of the tendon, which limited the range of motion of the wrist. Twelve cases were not satisfied with the ugly patchy scar of the donor site in the RFF flap group; they think it affects their confidence in daily social activities. No cases in the MSAP flap group did not reveal any complications, except linear scar in the donor site, which is totally acceptable. All the cases were satisfied (excellent, good) with the minimal donor site morbidity at the 12-month follow-up in the MSAP group; however, only 32% (8/25) of patients were satisfied (excellent, good) with the donor site morbidity postoperatively (Table 4). The MSAP flap group exhibited significantly better patient satisfaction (*p* < 0.001).

## Discussion

**Fig. 2:**
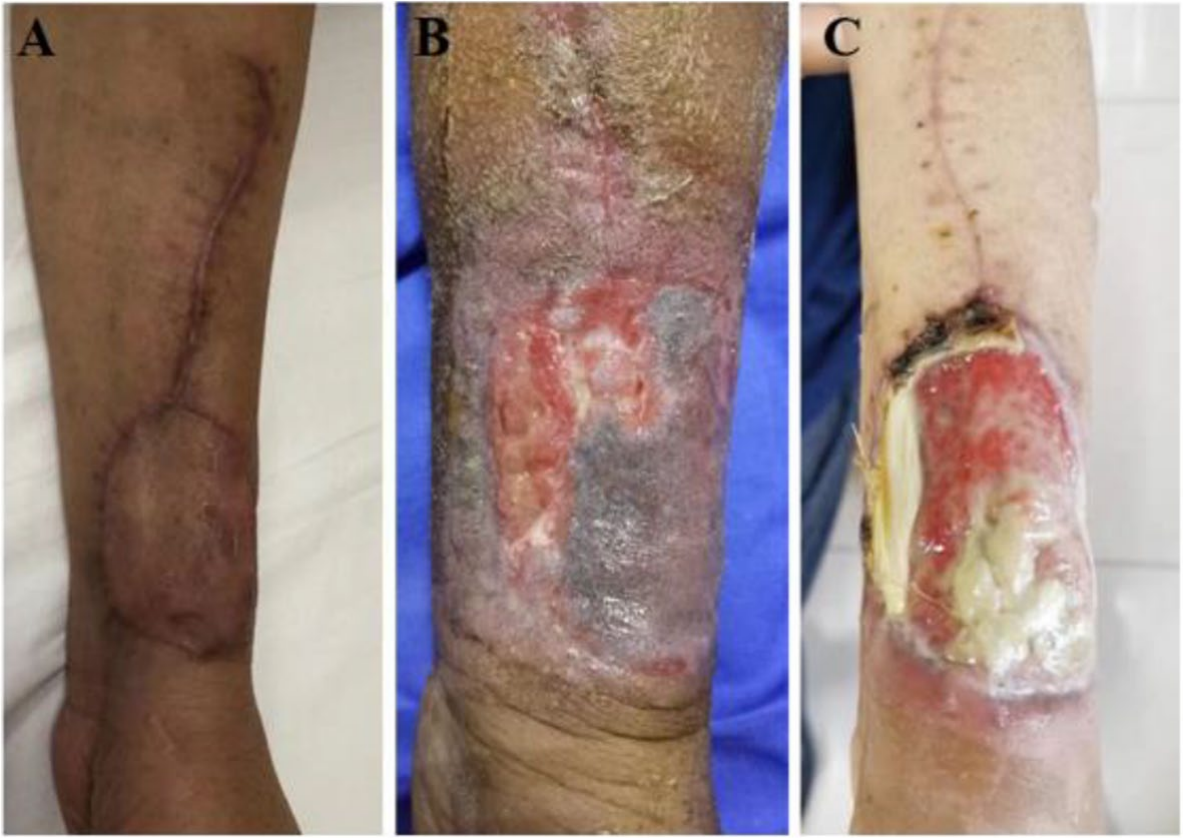
Significant donor site morbidity of RFF flap.** A** Ugly skin graft scar, **B** skin graft necrosis, and **C** tendon exposure

As microsurgery techniques have advanced, the use of free flaps has become common in head and neck reconstruction. The most popular flaps in head and neck reconstruction are RFF flap and ALT flap. The advantages of an RFF flap are that it is easy to harvest, thin, and reliable; therefore, it is always the first free flap that beginner surgeons can master. However, RFF flap has several disadvantages, such as sacrificing a main artery of the forearm, significant donor site morbidity, ugly skin graft scar, skin graft necrosis, and tendon exposure [4] (Fig. 2). As perforator flaps are becoming increasingly popular [7], ALT flap is used in the majority of head and neck reconstruction cases at present. The advantage of the ALT flap is its diversity of applications and that it provides more than enough tissue volume for reconstruction and poses minimal donor site morbidity. However, for medium-sized defects, especially during reconstructing buccal defects and those after semi-glossectomy, the ALT flap seems too bulky and provides too much soft tissue volume to properly restore tongue movement postoperatively. Some researchers [8, 9] have reported that ALT flap can be thinned, but thinning the flap inevitably increases the risk of flap necrosis. Additionally, the thinning process should be done at least 2–3 cm away from the perforators [10]. Apparently, it is not suitable for small- to medium-sized defects.

Since it was first described by Cavadas [11] in 2001, the MSAP flap has drawn attention of many microsurgeons. MSAP flap is a thin, pliable flap, has a long vascular pedicle, less hair-bearing skin, and enables easy dissection with minimal donor-site morbidity. It is also reliable, and there was only one flap necrosis occurred in the MSAP flap group despite only carrying one perforator in our study. Therefore, for a small-medium-sized flap, one perforator is enough for the whole flap blood supply. The survival rate of the MSAP flap in our study is 95.2%; similarly, many other surgeons [12–15] also reported a high survival rate of the MSAP flap. According to our study, the average pedicle length of the MSAP flap is 11.55 cm, which is longer than the average pedicle length (10.64 cm) of the RFF flap, but there is no significant difference (*p* = 0.068), both exceed the requirements of head and neck reconstruction [13, 16, 17]. The mean dimensions of the MSAP flap in our case series were 7.33 cm × 4.93 cm, which provides adequate tissue for coverage of medium-sized defects, like half-glossectomy or buccal defect. This is similar to the RFF flap group size, which is 7.44 cm × 5.12 cm (*p* = 0.787,* p* = 0.377).

**Fig. 3:**
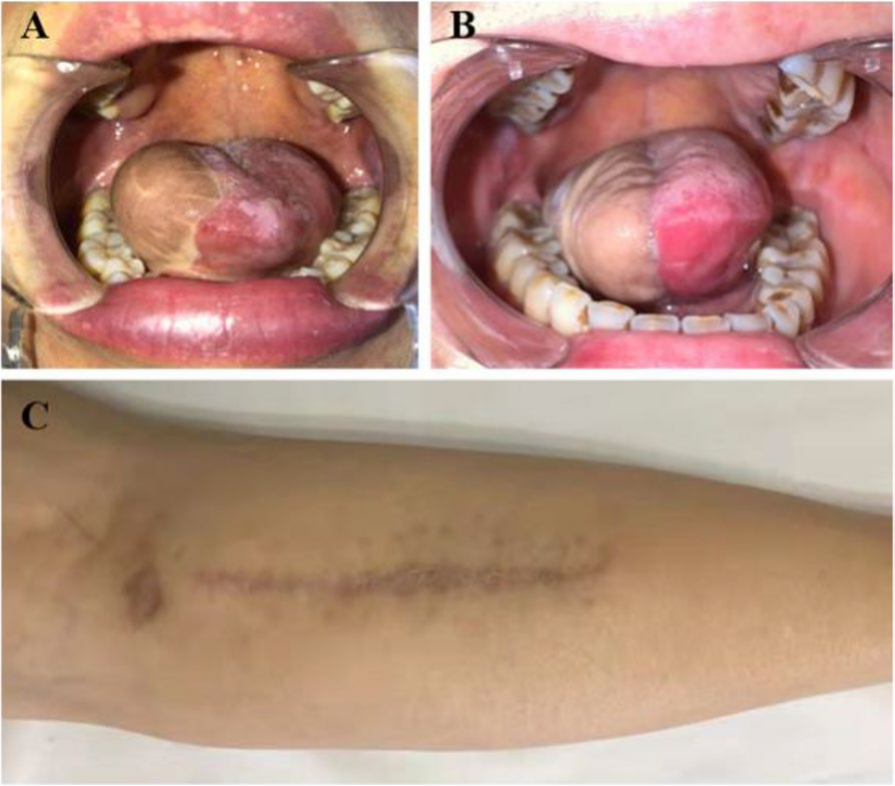
Postoperative morphology of donor and recipient sites of MSAP flap. **A** and **B** MSAP flap is better in maintaining the shape of the tongue. **C** The donor site in the MSAP flap

In our study, the flap thickness was 6.05 mm in the MSAP flap group, which seems thicker than the thickness in the RFF group (5.28 mm,* p* = 0.052), which is a suitable thickness for oral cavity reconstruction, but it did not show significant difference. In our experience, the MSAP flap is better in maintaining the shape of the tongue (Fig. 3A and B), but it is not as pliable as the RFF flap.

We selected patients with defects projected narrower than 7 cm preoperatively in our study, so that we could directly close the donor site postoperatively. Some researchers [12, 18] suggested that the width should be less than 5–6 cm to enable primary closure of the donor site. Daar’s [19] analysis revealed that a flap width greater than 5.75 cm would cause more donor site complications if not closed primarily. It would not completely show the advantages of the MSAP flap compared to those of the RFF flap. For patients with defects wider than 7 cm, other flaps are suggested for reconstruction, such as thinned ALT flap or deep inferior epigastric perforator flap. The donor site morbidity in the MSAP flap is a thin linear scar, which is hidden and acceptable (Fig. 3C). In contrast, the donor site in the forearm was frequently exposure site with ugly skin graft scar in the RFF flap group, which aggressively affects the patients’ confidence of participating in social activities. None of the patients in the MSAP flap group had any long-term complications, except for one patient who felt pain in the donor site leg occasionally within 3 months of the operation, which subsided spontaneously. None of the patients have reported any limitations in walking in daily life. Through assessing subjective function and cosmetic satisfaction with our self-made questionnaire, all the patients are satisfied with the minimal donor site morbidity in the MSAP flap group, which is much better than the RFF flap group (8/25, 32%). Kao [12] reported similar results.

The mean harvesting time of the MSAP flap is 46.05 ± 6.05 (range, 40 to 63) min, which is more than the mean harvesting time (30.92 ± 6.11 min, range 20 to 47) of the RFF flap group (*p* < 0.01), that is because dissecting perforator intramuscular consumes more carefulness and time. When the time of the skin graft is considered, the total time of harvesting flap and donor site closure of two kinds of flaps is almost the same to our experience. Meanwhile, there is no need to do another incision to graft skin in the MSAP group, which increases the donor site morbidity. The poor blood supply of donor site and improper motion of the forearm and hand postoperatively were considered the main factors affecting the healing of skin graft.

The postoperative oral function regards the comparison of swallowing and speech function recovery did not show significant difference (*p* = 0.547,* p* = 0.471). In terms of no significant difference in flap size and flap thickness, the postoperative oral function is theoretically consistent with our expectation.

All the cases in the RFF flap group suffered abnormal sensation in donor site, which is related to dysfunction of the superficial branch of the radial nerve. The superficial branch of the radial nerve consists of medial branch and lateral branch, which dominates the sensory function of the ulnar half of the dorsal thumb, dorsal index, long, radial half of the ring finger, and the radial dorsal thumb. We conventionally preserve the superficial branch of the radial nerve when harvesting RFF flap. But the nerve would be pressed for about 7 to 10 days after the skin graft, in order to assure the survival of the skin graft, which could be one of the factors causing the dysfunction of the superficial branch of the radial nerve. That is why all the cases complain the abnormal sensation of donor site. In our experience, the abnormal sensation might be mostly recovered in 2–3 years after surgery. But patients feel uncomfortable before their total recovery, which is not common in the MSAP group. Skin graft also resulted in tendon adhesion in 3 cases, which led to slight limited range of motion of the wrist joint compared to the normal wrist joint.

**Fig. 4:**
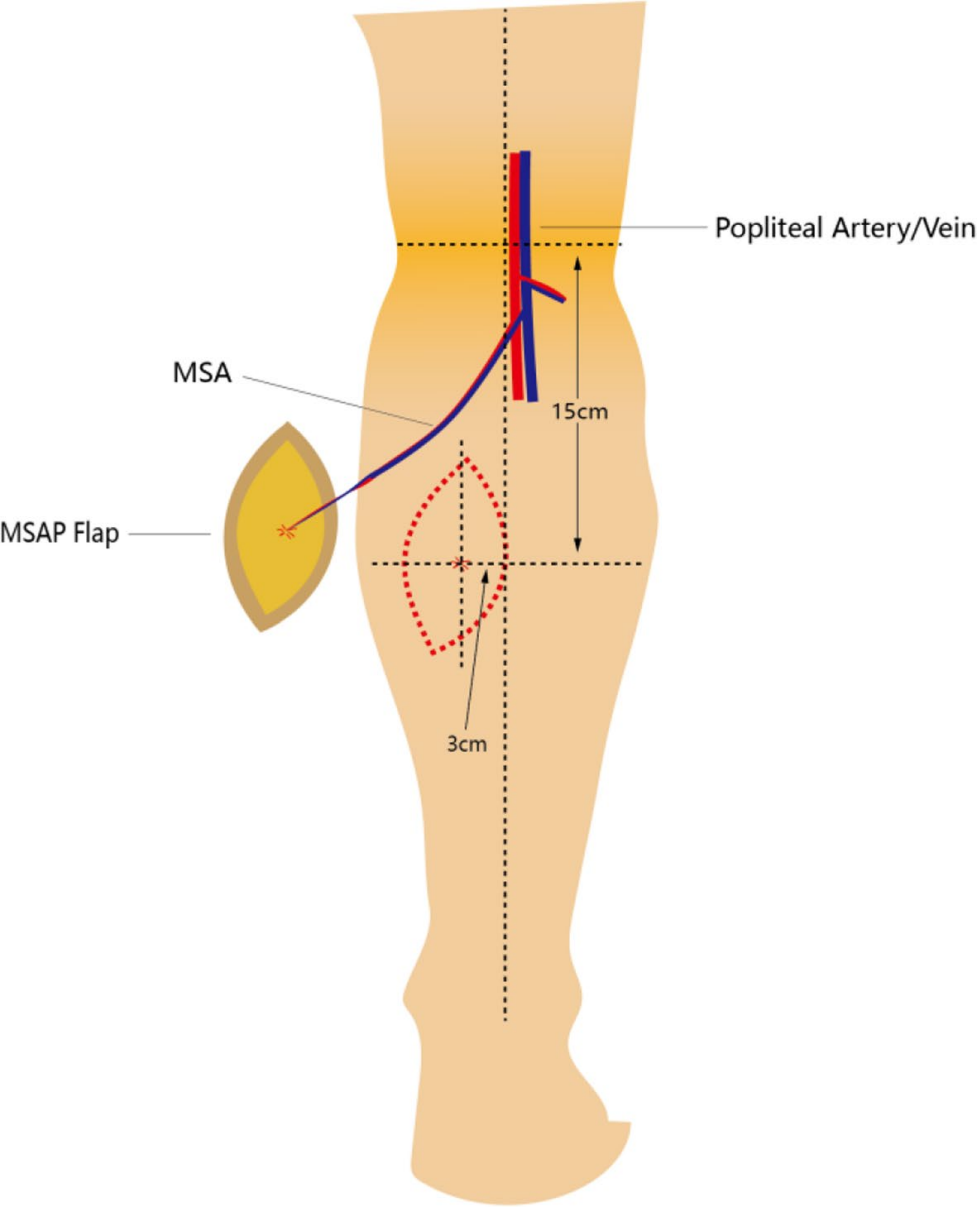
Schematic diagram of location of dominant perforator of MSA and MSAP harvesting

We regularly use color Doppler ultrasound to map the perforator preoperatively and design the flap according to the location where the dominant perforator emerges. This is reliable according to the comparison of the preoperative mark and the location where the perforators actually emerge intraoperatively in our study. So we believe the perforator mark of the other leg also was accurate. We can see the dominant perforator (Fig. 4) mostly emerges near the point which is 15 cm from PF vertically, and 3.15 cm from PML horizontally. This can guide us for flap designing even without perforator mapping preoperatively, which is similar to the research outcomes of Basnet L M [20], but we should leave a route of retreat in case of inaccuracy of perforator mapping. Some authors [21, 22] also have reported an opposite result and have pointed out that Doppler ultrasound is not a reliable technique. Meanwhile, some microsurgeons also locate the perforators using computed tomographic angiography or magnetic resonance angiography, but those need more time and are much more expensive compared to Doppler ultrasound, and their efficacy in mapping perforators is not precise enough. Inaccurate perforator location would misguide the flap design. In this case, the perforators should be explored intraoperatively, and the flap design should be adapted based on the actual location of the perforator. Therefore, we cannot rely completely on machines instead of carefulness.

Furthermore, MSAP flap also could form a chimeric flap [23] consisting of the conventional medial gastrocnemius muscle flap to reconstruct complex defects, such as floor muscle defect. Similarly, the muscle flap can refill the dead space of the mouth floor to avoid fistula. However, MSAP chimeric flap would increase donor site morbidity.

MSAP flap is reliable, has a suitable thickness of skin paddle, does not need a skin graft, and has minimal donor site morbidity for small- to medium-sized defects compared with RFFF. The question is why is the MSAP flap not as popular as RFF flap? There were three reasons, as mentioned below. First, many microsurgeons are not familiar with the anatomy of the perforator of the medial sural artery (MSA). The MSA and lateral sural artery (LSA) can come directly from the popliteal artery or common sural artery at the popliteal crease level. The MSA and LSA descend to the medial and lateral heads of the gastrocnemius muscle, respectively. Along its course, the MSA has three branching patterns. Type I (31%) exhibit a single main branch, Type II (59%) have a double branching pattern, and Type III (10%) have three or more branches. A dominant medial sural artery perforator can be identified in 92% of cases [24]; Dr.Deek [25] reported 200 consecutive cases of MSAP flap without the absence of the perforator. Also from the perforators’ data, we recorded and analyzed that a reliable MSAP flap can be harvested easily due to the relatively constant anatomy of perforators. Second, most perforators of the MSA are musculocutaneous perforators, which are easily injured intraoperatively, causing flap failure. Third, some surgeons think it is difficult to harvest the MSAP flap with the patient in the supine position as the original vessel of the pedicle is in the popliteal space. In our experience, the supine position with some rotation to the donor site side would enable the surgeon to complete the operation easily.

We present our study here aiming to popularize the MSAP flap in small- to medium-sized defect reconstruction. There might be other major perforators for MSA that need to be identified; we will do further research on them to extend the application of MSAP (chimeric) flap. Due to insufficient cases, further studies are required to accumulate more data and experience.

## Conclusions

The MSAP flap is reliable with a thin skin paddle and shows minimal donor site morbidity and better patient satisfaction compared with the RFF flap; meanwhile, there is no significant difference in postoperative oral function. It is therefore a suitable replacement for the RFF flap for reconstructing small- to medium-sized defects in the head and neck, although it needs consuming more time and dissecting cautiously. The perforator of the MSA is relatively constant.

## Supplementary Information

Additional file 1: Video.

## Data Availability

No datasets were generated or analysed during the current study.

## Abbreviations

MSAP: Medial sural artery perforator
RFF: Radial forearm free
AJCC: American Joint Committee on Cancer
UW-QOL: University of Washington Quality of Life Questionnaire
ROM: Range of motion
PF: Popliteal fossa
PML: Postor midline
ALT: Anterolateral thigh
MSA: Medial sural artery
LSA: Lateral sural artery
